# LoRa Scalability: A Simulation Model Based on Interference Measurements

**DOI:** 10.3390/s17061193

**Published:** 2017-05-23

**Authors:** Jetmir Haxhibeqiri, Floris Van den Abeele, Ingrid Moerman, Jeroen Hoebeke

**Affiliations:** Department of Information Technology, Ghent University—imec, IDLab, Technologiepark-Zwijnaarde 15, B-9052 Ghent, Belgium; floris.vandenabeele@ugent.be (F.V.d.A.); ingrid.moerman@ugent.be (I.M.); jeroen.hoebeke@ugent.be (J.H.)

**Keywords:** low-power wide area networks (LPWAN), LoRa, LoRaWAN, Internet of Things (IoT), scalability, interference modeling

## Abstract

LoRa is a long-range, low power, low bit rate and single-hop wireless communication technology. It is intended to be used in Internet of Things (IoT) applications involving battery-powered devices with low throughput requirements. A LoRaWAN network consists of multiple end nodes that communicate with one or more gateways. These gateways act like a transparent bridge towards a common network server. The amount of end devices and their throughput requirements will have an impact on the performance of the LoRaWAN network. This study investigates the scalability in terms of the number of end devices per gateway of single-gateway LoRaWAN deployments. First, we determine the intra-technology interference behavior with two physical end nodes, by checking the impact of an interfering node on a transmitting node. Measurements show that even under concurrent transmission, one of the packets can be received under certain conditions. Based on these measurements, we create a simulation model for assessing the scalability of a single gateway LoRaWAN network. We show that when the number of nodes increases up to 1000 per gateway, the losses will be up to 32%. In such a case, pure Aloha will have around 90% losses. However, when the duty cycle of the application layer becomes lower than the allowed radio duty cycle of 1%, losses will be even lower. We also show network scalability simulation results for some IoT use cases based on real data.

## 1. Introduction

The Internet of Things refers to the interconnection of devices such as sensors and actuators. Unlike the traditional Internet, the Internet of Things end devices are often power constrained and have low data rates to send to the network [1].

Low power wide area networks (LPWAN) are enabling wireless technologies that provide connectivity for the Internet of Things. This technology trades high bit rates for long range and low power. The bit rates provided by typical LPWAN technologies are in the order of hundreds of bits per second while battery life of the end devices can be as high as several years. Compared to multi-hop short-range technologies that were used until now for sensor networks, such as IEEE 802.15.4, LPWANs are single-hop networks where the end devices are connected directly to a gateway forming a star topology. A robust modulation technique ensures a wide coverage area with a single gateway, which can go up to several kilometers. Gateways will act as bridges towards IP-based networks. Typically, nodes will communicate only with a central server and not between each other. Another characteristic of LPWAN use cases is that the amount of uplink traffic exceeds the amount of downlink traffic, which is different from what we see in traditional cellular networks. Additionally, end devices can be connected even under low SNR conditions due to the usage of robust modulation techniques.

Different LPWAN technologies exist such as LoRa [2], SigFox [3], RPMA [4], NB-IOT [5], Weightless [6], etc. A comparison between ultra-narrow band (UNB) and spread spectrum technologies that are currently used in LPWAN is given in [7].

With the number of end devices increasing every day, with an estimate of 50 billion devices by 2020 [8] and an important part of them connected via LPWANs, understanding the scalability of such networks is important prior to end device deployment. In this paper, we will therefore study the scalability of LoRaWAN for scenarios with a single gateway deployment, which is the worst-case scenario. First, the impact of concurrent LoRa transmissions is assessed in order to create an interference model. The concurrent transmission impact is evaluated by means of a controllable setup consisting of real LoRa motes. We consider two different settings and change the SF, the preamble length and the received power at the receiver. Based on the outcomes of the measurements, we create an interference model. Through simulations based on the newly-created interference model, we show that an Aloha-like approach for assessing LoRaWAN scalability is not applicable due to its powerful physical layer. The majority of the simulation results assume a worst case scenario, where end nodes are sending as often as the physical layer duty cycle allows. Next to this, we also demonstrate the potential of the simulation model to assess the scalability in terms of number of devices per gateway for heterogeneous IoT applications with specific traffic needs.

First, in Section 2, related works are discussed. In Section 3, we will give an introduction to the LoRa technology and LoRaWAN. We further show the impact of concurrent LoRa transmissions in Section 4. We consider two scenarios: a scenario where the interfering transmission is received with the same RSSI as the interfered transmission on the gateway and a scenario where the interfering transmission is received with a higher RSSI compared to the interfered transmission. Based on the results from these real measurements, we create a model for simulating the scalability of large-scale single gateway networks. The simulation environment is introduced in Section 5. In Section 6, the results from simulations are covered for different scenarios with a 1% radio duty cycle, followed by a comparison between LoRa and pure Aloha. Since in IoT networks, the application layer update rates for specific applications can be lower than the 1% radio duty cycle limit, we also quantify the network scalability for different IoT application use cases. The simulations in this case will take into account real data about the number of end devices in a real city area. Section 7 discusses future work, whereas Section 8 presents the conclusions of this work.

## 2. Related Work

Since LoRa is a new technology, the amount of research on different aspects of both the LoRa technology and LoRaWAN networks is limited. To clarify the terms used throughout this paper: we will use LoRa to refer to the physical layer modulation itself, whereas we will use LoRaWAN to refer the deployment of LoRa based WANs using the LoRaWAN MAC protocol as standardized by the LoRaWAN Alliance [9].

In one of the first papers published on LoRa [10], the authors describe the advantages of LoRa over the other LPWAN technologies. They list the advantage of usage chirp modulation at the physical layer and the possibility of adopting upper layer solutions from other technologies such as IEEE 802.15.4 or 6LowPAN. In [11], the authors discuss the physical and MAC layer of different LPWAN technologies such as SigFox (ultra-narrow band (UNB)) and LoRa (chirp spread spectrum (CSS)). Theoretically, they calculated that the LoRa coverage can go up to 22 km compared to 63 km for SigFox. For LoRa self-interference, they calculated the co-channel interference rejection for all combinations of spreading factors (SF). They estimated that one transmission can be received with the same SF and in the same channel if it is received 6 dB higher than its interferer [11].

There have been a couple of works dealing with LoRaWAN coverage in different environments. In [12], the authors reported results for LoRaWAN coverage when deployed in a sub-urban area. They reached coverage up to 3 km. Based on simulations, they claim that the LoRaWAN MAC protocol is similar to pure Aloha in terms of scalability performance, meaning that the performance degrades quickly when the load on the link increases. In [13], the authors show the results regarding the data transfer for a single end device in LoRaWAN networks. They show that nodes near the gateway can send only 2 kbit/s in the uplink. They also show the possible end device distribution using different SFs, again assuming pure Aloha access for end devices. Our study proves that the LoRaWAN MAC protocol performs better than predicted by the pure Aloha channel access model in terms of collisions due to the robust LoRa physical layer. In [14], the authors give an introduction to LoRaWAN networks and coverage planning for such networks. They achieved planning network coverage for a city of 100 km${}^{2}$ with only 30 gateways, half the number of base stations currently required for cellular network deployments in the same area. The LoRaWAN performance when deployed in an indoor environment was studied in [15]. Here, the authors made excessive measurements to characterize the performance in terms of packet loss, indoor coverage, received signal strength at gateways, power consumption of end devices and delays due to radio duty cycle. Indoor LoRaWAN coverage was studied in [16], as well. Another LoRaWAN coverage study was presented in [17]. Based on real measurements, they achieved up to an 80% packet success rate for distances lower than 5 km from the gateway and a 60% success rate for distances from 5 to 10 km. Contrarily, on open sea, they reached up to a 70% packets delivery ratio for a distance of 15 km, which is quite promising. Based on those real results, they model the propagation channel for LoRa technology [17].

Two methods for decreasing the inter-network interference and for improving the reception rate are the usage of directional antennas and the usage of multiple base stations. The impact of these two methods in decreasing the inter-network interference in LoRaWANs is studied in [18]. Using simulations, the authors show that for the multiple base stations’ case and having nodes with omni-directional antennas, the data extraction rate is 56% compared to only 32% when directional antennas are used.

A LoRaWAN scalability study is presented in [19,20], where the authors developed a mathematical model of the transmission process to assess the scalability of LoRaWAN. They concluded that the network capacity is only 0.1 51 byte frames per second. This capacity corresponds to 5000 motes each transmitting two messages per day [19]. This capacity is for confirmed uplink traffic. In our work, we take a different approach, assessing the scalability of a LoRaWAN network where end nodes send as often as the radio duty cycle allows. This assessment gives a lower bound of the maximal number of nodes that can be served by a single gateway. We consider unconfirmed uplink traffic, but take into account the radio duty cycle limitations.

Another scalability study is presented in [21]. They based their simulation model on the outcome of measurements with real nodes, but their measurements are not done under an interference-free environment. They take into account only three specific parameter settings for nodes. We will take into account all of the combinations of the possible spreading factors and three mandatory channels at the 868 MHz band. Moreover, we will show the scalability for different IoT applications use cases. In [22], a scalability study for LoRaWAN based on a stochastic geometry framework is presented. They show that the coverage probability drops exponentially with the increase of end device numbers.

In [23], the authors made a study regarding the CSS modulation technique. They show that not any two CSS symbols are always orthogonal. Based on simulations, they show that the achievable range of the CSS modulation technique is lower than an ultra-narrowband solution, but the robustness against interference is higher.

Apart from researchers, even regulatory authorities are doing their own studies regarding the spectrum usage and interference impact of different technologies based on LPWAN deployment in sub-GHz bands. Such a study is reported in [24].

## 3. Introduction to LoRa and LoRaWAN

The LoRa technology can be separated into two parts: the physical layer, which is patented by Semtech [25], and the MAC layer protocol and network system architecture, called LoRaWAN, designed by the LoRa Alliance [9]. The physical layer uses spread spectrum modulation and forward error correction techniques to make the communication robust against noise and interference and to increase the receiver sensitivity.

The physical layer operates in the 433-, 868- or 915-MHz frequency bands. In Europe, only the 868- and 433-MHz bands can be used. In the 868-MHz band, there are three 125-kHz channels that are mandatory to be implemented in every end device. There are another five 125-kHz channels in the 867-MHz sub-band that can be optionally used for LoRa communication [26]. We will limit our study to the usage of the three mandatory channels, which forms the minimum set of channels that each network gateway has to listen on. Due to European transmission regulations [27], each transmission in any of those three channels should comply with a 1% radio duty cycle in the case that there is no listen-before-talk and adaptive frequency agility mechanism implemented. This means that if the radio transmitted for 1 s, then it cannot transmit for the next 99 s.

Each bit of information is represented by multiple chips of information. By increasing the spreading factor, the number of chips per symbol is increased, thereby decreasing the nominal data rate. Six different spreading factors are used, from seven to 12, that are orthogonal to each other [25]. The number of chips per symbol is calculated as 2${}^{SF}$. To improve the robustness of the communication, forward error correction (FEC) codes are used with a coding rate from 4/5 up to 4/8. Diagonal interleaving is used to increase the robustness against long interference. With diagonal interleaving and coding rate of 4/7 or 4/8, up to +1/−1 position errors can be corrected, compared to single error correction if row-interleaving is used [25].

The packet structure at the physical layer includes a preamble, an optional header and the data payload. The preamble is used to synchronize the receiver with the transmitter and can have a length from 10 up to 65,536 symbols in total. The fixed part of preamble consists of four symbols, and the rest is programmable with a minimal length of six symbols and a maximal length of 65,532 [28]. According to [25], the preamble starts with a sequence of constant upchirp symbols that is programmable and helps to detect the start of the frame. The programmable part is followed by two upchirp symbols encoding the sync word that is used for frame synchronization. The sync word can also be used to distinguish between devices from different networks. If the sync word does not match the sync word that is configured on the gateway, then the gateway will stop receiving that packet. Finally, the preamble ends with two downchirp symbols that are used for frequency synchronization. After the last two symbols, a 0.25 symbol time represents a silence time used to let the receiver align the time [25].

The header can be implicit or explicit. In the latter case, the header contains the payload length in bytes, the FEC code rate of the payload and the header CRC. The header is always protected with the FEC of the highest code rate of 4/8. If all of these three parameters are known in advance, the header can be removed completely. This decreases the time on air of the packet. In this case, the implicit header mechanism is applied, where the header parameters are fixed beforehand at both the receiver and the transmitter side. The payload contains either LoRaWAN MAC layer control packets or data packets. Optionally, it can be followed by the payload CRC.

The LoRaWAN MAC layer provides the medium access control mechanism that enables the communication between multiple devices and their gateway(s).

The LoRaWAN network architecture has a star topology, where the end devices can only communicate with LoRaWAN gateways and not directly with each other. Multiple gateways are connected to a central network server. The LoRaWAN gateways are only responsible for forwarding raw data packets from end nodes towards the network server. The network server is responsible for sending downlink packets towards end devices, if needed. The LoraWAN standard defines three classes of end-devices. The features of Class A devices are basic sets of options that every end device needs to implement in order to join a LoRaWAN network. In order to enable bidirectional communication, each uplink transmission of a Class A device is followed by two short downlink receive windows during which the end device will listen for possible downlink traffic. Therefore, the downlink communication is triggered by the end device, meaning that the network server first needs to wait first for an uplink communication. The first and second downlink window start at 1 and 2 s, respectively, after the end of the uplink transmission. It is the responsibility of the network server to schedule the downlink traffic at the exact time and to perform the timing control. Class A end devices consume the least power since most of the time, they are asleep. We used only Class A devices during our study. For other classes of end devices, we refer the reader to the LoraWAN standard document in [9].

## 4. Interference Measurements with LoRa Nodes

With an increasing number of nodes per gateway, the number of concurrent LoRa transmissions will increase, as well, increasing the probability of collisions and hereby affecting throughput. In this section, we will experimentally prove that, since LoRa uses a robust modulation scheme, it is possible that one packet that is part of concurrent transmissions can still be received correctly. This depends on the start time of the collision, as well as the interferer strength as seen from the gateway. This is contrary to traditional Aloha-like network modeling, where it is assumed that once part of the packet collides with another packet, both packets will be lost.

**Figure 1:**
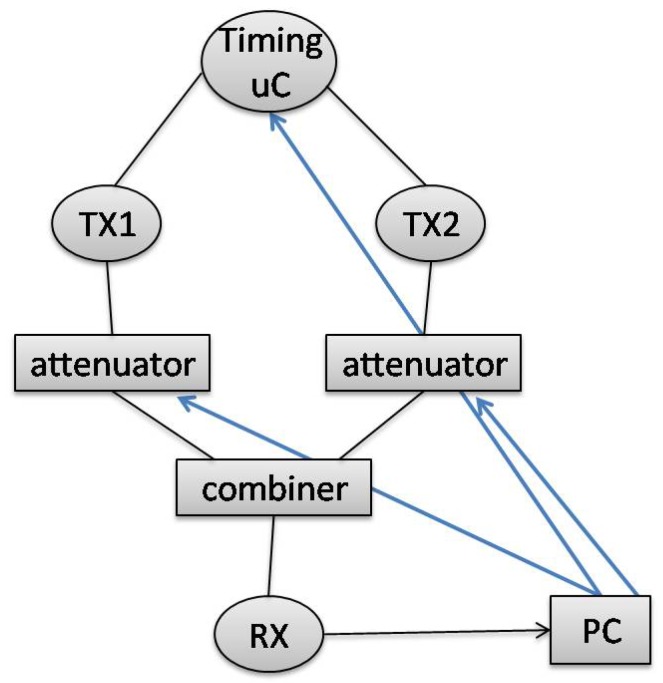
Controllable setup for measurements with real nodes.

In order to quantify the impact of intra-interference in LoRa networks and how the interference will affect the number of nodes served by gateway, we used a controllable setup as shown in Figure 1. We used two LoRa transmitters and a LoRa gateway. Both transmitters are connected to a microcontroller, which will control the transmission timings of both transmitters. A tunable attenuator between each transmitter and a combiner is used to change and decrease the RSSI at the receiver side, while the combiner will combine the signals towards the receiver. A PC is used to control the attenuation on the paths, to program the microcontroller, as well as to collect the data from the gateway once the measurements are finished.

The setup was deployed inside RF shielded Qosmotec boxes in order not to experience any interference from other transmissions in the environment, as well as not to interfere the external environment during our measurements campaign. The attenuation box was of type PAH-6000/80-2 with eight input/output ports with maximal insertion losses of 14 dB. The splitter/combiner box was from MTS Systemtechnik with eight paths with maximal insertion losses of 6 dB. The documentation for the Qosmotec RF shielded boxes, attenuation box, as well as splitter/combiner box can be found in [29,30,31], respectively.

We used two IMST iM880A [32] nodes as LoRa transmitters and a LoRank gateway, which uses the WiMode iC880A [33] concentrator module with the SX1301 [34] digital baseband chip. On the gateway, a packet logger application was running, which collected data including SNR and RSSI values for all received packets and data packets together with metadata information, such as the spreading factor used, channel used, coding rate used and received payload CRC. For the measurements, we used the physical layer parameters as indicated in Table 1, with two different settings. Table 1 also shows the timings for such a configuration. The on-air time of a packet was 1712 ms and 76.03 ms, respectively, and the preamble length of the packet was 401.41 ms and 18.69 ms respectively.

**Figure 2:**
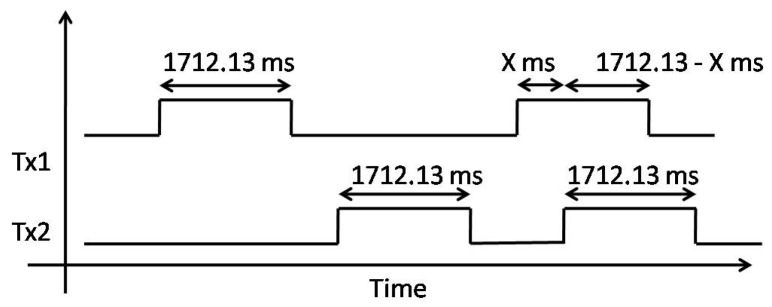
Timing diagram for one measurement period for the first setting.

In order to speed up the measurements, we disabled the radio duty cycle on the transmitter nodes. Measurements were done periodically, meaning we had an uncollided slot for each transmitter, followed by a third slot during which a collision of both transmissions occurred. We did measurements for 15 min, resulting in around 200 uncollided packets and 200 collided packets for every transmitter. In Figure 2, the timing diagram for one period for the first setting is given, the same period being repeated for 15 min.

The transmission of the interferer during the collision slot was delayed from 100 up to 1700 ms after the start of the first transmission with a step of 100 ms (X in Figure 2) for the first setting and from 10 up to 70 ms for the second setting respectively with a step of 10 ms. This was done to check the impact of collisions on different parts of the packet, as we assumed that the importance of the preamble and the payload would not be the same.

In the next two subsections, we give the results for two cases. The first one is where the interferer has the same RSSI as the interfered transmitter at the receiver. The second one is where the interferer has a higher RSSI than the interfered transmitter.

### 4.1. Interferer Received with the Same RSSI as the Interfered Transmission

In this case, the attenuation on both paths between transmitters and receiver was the same, namely 100 dB. The transmit power of both transmitters was set to 14 dBm. Together with the insertion losses of the ports of attenuators and the combiner, the total attenuation was summed to 120 dB ± 2 dB. The RSSI values at the receiver for both transmitters were −110 dBm ± 2 dBm.

**Figure 3:**
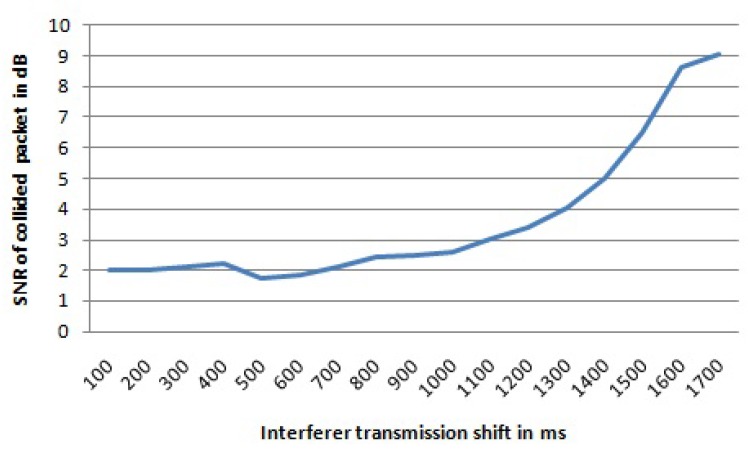
Average SNR value for received packets from the interfered transmitter for the first setting.

In Table 2, the statistics of the collided packets for the first setting from both transmitters are shown, while Table 3 shows statistics for the second setting. The interfered transmitter is referred to as Tx1, while the interferer is referred to as Tx2. Packets lost due to a collision refers to packets that have never been received, whereas packets received with bad CRC refers to packets that have errors, but that cannot be corrected. When the packet is collided and is lost, the gateway is not able to report any data on the SNR value, while for packets received with wrong CRC the SNR value is reported accordingly. First, when the interferer transmission starts only 100 ms after the interfered transmission, 25% of the collided packets from the interfered transmitter are received with a wrong CRC in their payload, albeit the packet losses were low, only 2%. By delaying the interferer transmission for more than 300 ms after the start of the interfered transmission, most of the collided packets from the interfered transmitter are received with a correct payload CRC. The number of packets received with a wrong payload CRC is less than 5%, except for the case when the interferer transmission is shifted for 500 ms and 800 ms, respectively. However, the sum of lost packets and packets received with wrong payload CRC can be up to ∼10% (for an 800-ms shift). Similar behavior was noticed for the second setting. When the shift was smaller than the preamble length of 18.69 ms, there were packets received with a wrong payload CRC. For shifts higher than the preamble time, most of the packets were received correctly, although the percentage of losses augmented with the percentage of packets received with wrong payload CRC can be as high as 7.7% (for a 30-ms shift). The average SNR value of all received packets as a function of the shift of the interferer transmission is given in Figure 3 for the first setting. By delaying the interferer transmission, the average SNR value of the packets received from the interfered transmitter is increased. In this case, the collision time becomes shorter, and as consequence, the SNR value of the interfered transmission increases.

On the other hand, all packets from the interferer transmitter are lost when the interferer transmission is delayed less than 1400 ms (70 ms) from the interfered transmission. This is due to the fact that the preamble of the interferer transmission (∼400 ms, (18 ms)) always collides with the interfered transmission, and the receiver cannot synchronize with the interferer preamble since it is already synchronized with the previous transmission. In this case, the receiver will not resynchronize with the interferer transmitter as the RSSI of the interferer transmission is in the same range as the interfered transmission, and it will only be seen as noise for the interfered transmission. In case the delay of the interferer transmission is more than 1400 ms (70 ms) from the start of the interfered transmission, then the interferer transmission will be picked up from the receiver too in addition to the interfered one. As the preamble time length is 401.41 ms, then, for the case when the interferer transmission is delayed for 1500 ms, the last 189.28 ms (401.41 to (1712.13 to 1500) ms) of the preamble of the interferer transmission are uncollided. This time duration results in six uncollided symbols of the preamble. As such, the receiver can synchronize on the last symbols of the preamble and receive the interferer transmission correctly. For the last two shifts of the interferer transmission, more than 75% and 94% packets are received correctly for the first setting, respectively. The same thing happens with the second setting. For a shift of 70 ms, the last six symbols of the preamble of the interferer do not collide, and the receiver can receive the packet accordingly, resulting in 100% of packets being received correctly.

Based on these results we can conclude that if the interferer starts after the preamble and the RSSI from the interferer is at the same level or lower than the interfered transmission, then the interfered transmission will be received correctly. Furthermore, it suffices that at least the last six symbols of the preamble have to be received without any collision in order for the receiver to synchronize with the transmitter.

### 4.2. Interferer Received with Higher RSSI as the Interfered Transmission

In this case, the attenuation on the paths between transmitters and receiver was different. On the path between the first transmitter and the receiver, the introduced attenuation was 100 dB, whereas on the path between the second transmitter and the receiver, it was 90 dB. The transmit power for both transmitters was set to 14 dBm. Together with the insertion losses of the ports of the attenuators and the combiner, the total attenuation summed up to 120 dB ± 2 dB for the first path and 110 dB ± 2 dB for the second path. The RSSI value at the receiver was −110 dBm ± 2 dBm for the interfered transmitter and −100 dBm ±2 dBm for the interferer transmitter. Therefore, the RSSI difference between the interferer and the interfered transmissions was 6 to 14 dB. The interferer has a stronger signal at the receiver than the interfered signal.

In Table 4, the statistics of the collided packets for the first setting from both transmitters are shown, while Table 5 shows the statistics for the second setting. The interfered transmitter is referred to as Tx1, whereas the interferer is referred to as Tx2. For the first three shifts (100, 200 and 300 ms), packets from both transmitters were lost. The same behavior is observed for the second setting for shifts of 5 and 10 ms. All of these shifts, for both settings, happen during the preamble time of the interfered signal. As the interferer transmitter has a higher RSSI at the receiver, it makes the synchronization of the receiver with the interfered transmitter to be lost. As such, when the interferer starts during the preamble time, there is no recapture effect. However, the recapture effect is only seen for the shifts of 400 and 500 ms, for the first setting, and 20 ms for the second setting, respectively. These shifts happen during the physical header time. Since the physical header has its own CRC, the receiver will stop receiving a packet in case the header CRC is wrong. For these shifts, the receiver will stop receiving the interfered transmission (due to the wrong header CRC), and as such, it is able to re-synchronize with the interferer transmission and can receive it correctly. However, for shifts higher than 600 ms (20 ms for second setting), that is higher than the preamble plus header time, the receiver will continue to receive the interfered transmission, as it is already synchronized with it and the header was received correctly. Consequently, for shifts greater than 600 ms (20 ms), no recapture effect will happen, even when the interferer transmission has a higher RSSI at the receiver side than the interfered transmission. As the interferer RSSI is higher, this will lead to received packets from the interfered transmission having a wrong payload CRC. In [35] they report a similar effect, but they do not distinguish from which transmitter the packets with the wrong payload CRC were being received. Even for higher differences in RSSI values between the interferer and the interfered transmission (up to 100 dB), we did not notice any recapture effect in case the shift was greater than the preamble and header time. In Table 4 and Table 5, data for an RSSI difference of 12 dB are shown.

**Figure 4:**
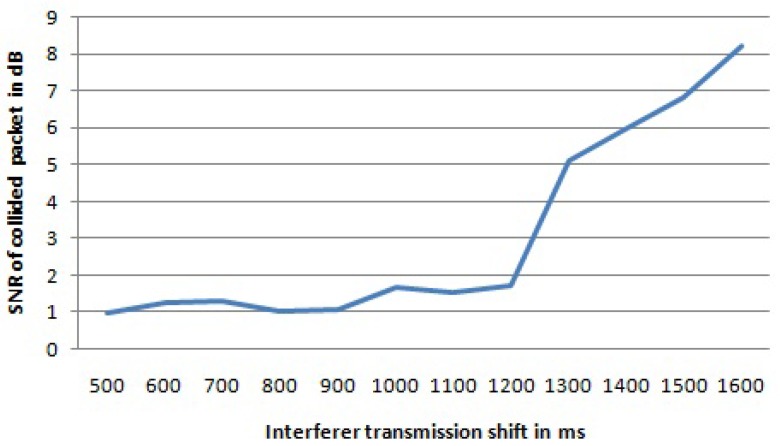
Average SNR value for received packets from the interfered transmitter for the first setting. For the first four shifts, there are no data since all the packets were lost.

By increasing the delay of the interferer transmission, the average SNR value for the received packets from the interfered transmitter will be increased. For the first four shift values (up to 400 ms), we do not have data for the SNR value because all packets from the interfered transmitter were lost due to collision. For shifts from 500 to 1200 ms, the average SNR value for the received packets stays under 2 dB, while for higher shifts of the interferer transmission, the average SNR value becomes higher. For bigger shifts of the interferer, the collision time becomes shorter, and as consequence, the SNR value of the interfered transmission increases. The graph with the average SNR values for the received packets of interfered transmissions for different shifts of the interferer transmission for the first setting is shown in Figure 4.

The interferer transmission will start to be picked up just when the shift is higher than 1400 ms for the first setting. Compared to the previous case, now for the same 1400 ms shift, only 48% of the packets from the interferer transmitter are lost compared to 84% in the previous case. This is due to the fact that the average SNR value of the interferer is higher than in the previous case. For shifts of 1500 and 1600 ms, there are no losses for packets coming from the interferer transmitter. For the second setting, the packets from interferer will be received correctly for shifts larger than 70 ms. Even here, the same explanation as in the previous subsection case holds. It suffices that the last six symbols from the preamble are received without any collision in order for the receiver to become synchronized with the transmitter.

Based on these results, we can sum up that if the interferer starts after the end of the preamble and header time and it has a higher RSSI value at the receiver, the interfered transmission will be received with the wrong payload CRC and that in case the last six symbols of the transmitter preamble are received correctly, then the receiver can be synchronized with the transmitter.

### 4.3. Measurement Conclusions and Generality

**Figure 5:**
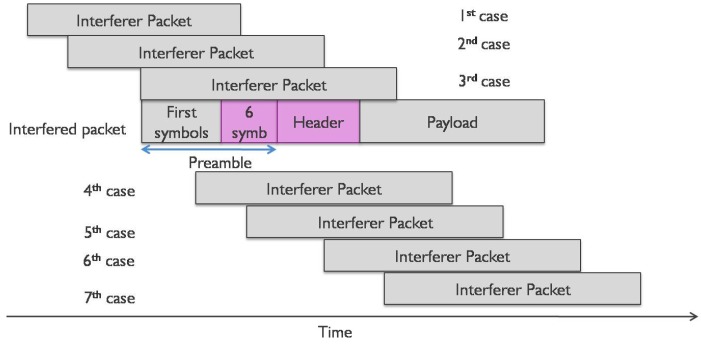
All of the possible cases that an interfered transmission can collide with the interferer.

In Figure 5, we show all of the possible interfered positions, whereas in Table 6 for each case, depending on the interferer RSSI value at the receiver, we show whether the packet is received correctly, classified as lost or received with the wrong payload CRC according to the measurements. The last column shows how we will classify the packet in our model that will be discussed in Section 5.

Based on the results from the real measurements, we can make the following conclusions:

- if the interferer starts after the preamble and the RSSI from the interferer is at the same level or lower than the interfered transmission, then the interfered transmission will be received correctly;
- if the interferer starts after the end of the preamble and the header time and has a higher RSSI at the receiver, then the first transmission will be received with the wrong payload CRC;
- if the last six symbols of the transmitter preamble are received correctly, the receiver can synchronize with the transmitter.

These conclusions are independent of the preamble length. The last six symbols correspond to the four fixed symbols and the last two symbols of the programmable part. According to [25], the length of the programmable part of the preamble is used to deal with receivers that have to go from a low power state to a fully-awake state, while the fixed part is used for synchronization. However, since we are considering only uplink traffic and the gateways are continuously in a fully-awake state, there is no need for long preambles. In such a case, the increase in preamble length will increase the power consumption of the end nodes and will not contribute to a higher reception probability. Since the gateway is fully awake, it needs few symbols in the programmable part to detect the start of the frame. We anticipate that in case other bandwidth channels are used (other than 125 kHz), we need to conduct additional experiments. However, in our simulation model, we only use the 125-kHz channel bandwidth, the most common one for deployed LoRaWAN nodes. Regarding the code rate impact, as we used the code with the highest coding rate (CR = 4) during the measurements, the usage of a lower coding rate (CR = 1, 2, 3) will not have any impact on the second case (already all of the packets from the first transmitter were received with the wrong CRC, and lower coding rates are less good at correcting errors). In the first case (i.e., interferer with the same RSSI as the interfered signal at receiver), the percentage of received packets with the wrong CRC from the interfered transmitter was rather small (∼5%) for shifts bigger than the preamble and header time. In case codes with lower coding rates (CR = 1, 2, 3) are being used, we expect this number to increase. However, our model will be conservative, as can be seen in Table 6 and will not be affected by the increased percentages of packets received with the wrong payload CRC. One case not covered by the measurements is the impact of multiple simultaneously transmissions. In such a configuration, there might be a case when there are multiple transmissions that contribute to the noise level that can make the reception of main transmission impossible. This is outside the capabilities of the setup.

## 5. Interference Modeling

Using the measurements with real nodes as input, as well as the determination of collision behavior, we created a simulation model for determining the number of end devices that can be served with a single LoRaWAN gateway.

We generate a vector of spreading factors (SF) used by each transmitter SF[*i*], with *i* = 1, ..., *N* and *N* the total number of transmitters served by the gateway. The vector is populated randomly with values for SF, ranging from seven to 12. We used the Hata–Okumura [36,37] propagation model for medium cities to determine the total area served by a single LoRa gateway. The height of the gateway was 25 m, and the height of the end devices 2.5 m. Using the sensitivity ranges for each SF, we determined the area covered by each SF if the transmit power of each node is the highest one allowed, namely 14 dBm (in the 868-MHz band). Sensitivity ranges, distance ranges and the percentage area covered by a single SF are given in Table 7. It can be noticed that based on the sensitivity range, the area covered by SF 12 is larger than the area covered by SF 7. Consequently, the number of nodes with SF 12 will be higher than the number of nodes with SF 7, assuming a uniform distribution of nodes in the area. Therefore, we made sure that the number of specific SF values in the SF vector matches the percentages shown in Table 7, e.g., for each random generation of the SF vector, we have around 22% transmitters using SF 12, 18% transmitters using SF 11, and so on. Therefore, the generation of SFs will stick to the percentages of the number of end nodes that is able to use a specific SF into the total population of the end nodes.

Next to this, we generate a second vector of RSSI values at the receiver RSSI[*i*], with *i* = 1, ..., *N* and *N* the number of transmitters served by the gateway. In reality, the SF that will be used by the end node is related to the RSSI at the gateway for that end node. When an end node is far away from the gateway or its signal is highly attenuated, the RSSI will be low, consequently forcing the end node to use a higher SF. As such, to make it realistic, the values of the RSSI vector are correlated to the values of the SF vector sensitivity ranges from Table 7.

We further generate a channel vector CHAN[*i*], with *i* = 1, ..., *N*. Since we only use three 125-kHz channels from the 868-MHz band for data communication in the LoRaWAN network, the values of the channel vector will be randomly chosen from the interval [1,3]. There are another 5125-kHz data channels at the 867-MHz sub-band, but only the first three channels at 868 MHz are mandatory for each Class A device.

As a last step, we generate the packet transmission start time matrix Time[*i*][*j*], with *i* = 1, ..., *N* and 0 ≤*j* < *n*, *N* the number of transmitters served by the gateway and *n* the number of packets that each transmitter has to send. We assume that *n* is the same for each transmitter during the tests. In order to respect the 1% duty cycle of the physical layer, two consecutive packet transmission start times are separated at least by a time difference of (τ× 100 − τ) seconds, with τ the on-air time of the previous transmission. τ depends on the SF used, the preamble symbol length, header type and the payload length. All *n* packets will be sent “as soon as possible”, meaning that the transmission start time of the *n*-th packet will be:

(1) $$t_{n}=\sum_{j=1}^{n}100\times \tau_{j-1}+\sum_{j=0}^{n}\delta_{j}$$

with τ the on-air time of the packet and δ is a random number in the range of [0, τ] that introduces some randomness with respect to the timings at which transmissions take place. Thus, we prevent that all of the transmission to happen at exact same time, but to have some randomness between each other. This way, the radio duty cycle of 1% is respected, but at the same time, the application update rate is maximized.

In order to determine the number of collisions and the number of packets received with the wrong payload CRC, we proceed as follows:

- For each transmitter *i* = 1, ..., *N* and *N* the number of transmitters served by the gateway, iterate through all other possible interferers *k* (*k* < *N* & *k* ! = *i*).
- If (SF[*i*]==SF[*k*]) and (CHAN[*i*]==CHAN[*k*]) check the starting time of the transmission by *k*.
- Check if the starting time of the interferer *k* occurs before the end time of the preamble and the header of the transmission by *i* or if the transmission of interferer *k* ends after the last six preamble symbols of the transmission by *i*. If these conditions hold, a collision has occurred as the interferer interfered the preamble and header of the transmission of *i*.
- Else, check if the start time of the interferer *k* is after the end of the preamble and the header of the interfered transmission. If the RSSI of the interferer is higher than the interfered transmission, the packet is received with the wrong payload CRC.

This procedure is shown in a more structured way in Algorithm 1.
**Algorithm 1:** Algorithm to determine the packet collisions and packets received with the wrong CRC.
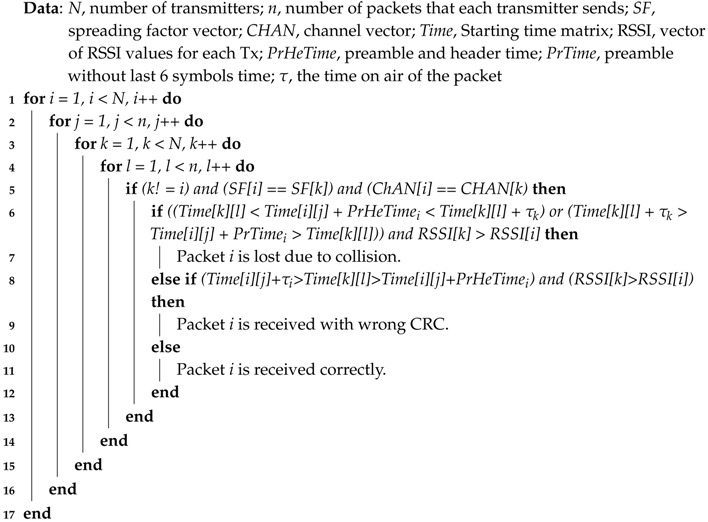


As such, in the simulation, we made a conservative model taking into account the worst subset of conditions from the outcomes of the measurements in Section 4.3. Therefore, in the simulation model, we covered Outcomes (i) and (ii) from our measurements in one condition by checking if the interferer starts after the preamble and header time. In Table 6, we show how the packets are considered in our model based on different cases from Figure 5.

The input parameters for the simulation model are: *N*, the number of transmitters served by the gateway, *n*, the number of packets to be sent by each transmitter, and *K*, the number of tests to run for each case.

The assumptions of our simulation model are as follows: we assume that all transmitters send packets with the same payload length; we assume that transmitters do not switch the spreading factor from one packet to another during one test; we assume that the transmitters do not change the transmit power from one packet to another during one test; and we assume that all of the transmitters have the same number of packets to send.

The percentage of lost packets due to collisions and the percentage of the wrong payload CRC received packets for each test are calculated. The percentage of lost packets due to collisions for a single test *k*, *C*${}_{k}$, is given as:

(2) $$C_{k}=\frac{\sum_{j=1}^{n}\sum_{i=1}^{N}c_{ij}}{nN}\times 100$$

where:

(3) $$c=\left\{\begin{array}{cc} 0, & \mathrm{if}\;\mathrm{packet}\;\mathrm{was}\;\mathrm{not}\;\mathrm{collided}\\ 1, & \mathrm{if}\;\mathrm{packet}\;\mathrm{was}\;\mathrm{collided} \end{array}\right.$$

The total percentage for each case will be given as the average of tests’ percentages:

(4) $$C_{tot}=\frac{\sum_{k=1}^{K}C_{k}}{K}$$

The same is valid for the number of packets received with the wrong payload CRC. The percentage of packets received with the wrong payload CRC per test, BAD_CRC, is given as:

(5) $$BAD\_CRC_{k}=\frac{\sum_{j=1}^{n}\sum_{i=1}^{N}bad\_crc_{ij}}{nN}\times 100$$

where:

(6) $$bad\_crc=\left\{\begin{array}{cc} 0, & \mathrm{if}\;\mathrm{packet}\;\mathrm{was}\;\mathrm{received}\;\mathrm{correctly}\\ 1, & \mathrm{if}\;\mathrm{packet}\;\mathrm{was}\;\mathrm{received}\;\mathrm{with}\;\mathrm{the}\;\mathrm{wrong}\;\mathrm{payload}\;\mathrm{CRC} \end{array}\right.$$

The total average percentage of packets received with the wrong payload CRC for each case is given as:

(7) $$BAD\_CRC_{tot}=\frac{\sum_{k=1}^{K}BAD\_CRC_{k}}{K}$$

The total average percentage of lost packets will be the sum of the total average percentage of lost packets due to collisions and the total average percentage of received packets with the wrong payload CRC.

In order to make a comparison with a pure Aloha network, we created another simulation model to calculate the scalability of pure Aloha. The model also took into account the 1% radio duty cycle of the 868-MHz band. A collision was detected once the slice of packet collided with another packet. No statistics of packets received with the wrong payload CRC were calculated, since in the pure Aloha case, the packet is either received correctly or lost due to collision. The input of the pure Aloha simulation model is the same as for the LoRa simulation model. The calculated outputs are also the same, except for the calculation of packets received with the wrong CRC, which do not occur.

The reason why we are distinguishing between lost packets due to collisions and packets received with the wrong payload CRC in LoRa is that the latter case means that the receiver achieved correctly receiving the preamble and the header. As such, the receiver at least achieved synchronizing with the transmitter, and only the payload was received with errors.

## 6. Simulation Results

In this section, we will give the results of the scalability simulations based on the simulation model described in Section 5. For the simulations, we used six different payload lengths: 10, 20, 30, 40 and 50 bytes. The number of transmitters varied from one up to 1000 transmitters per gateway. Each transmitter sends 10 packets per test, and every test was repeated 100 times. For the case of a single channel and single SF simulations, only one channel out of all three possible channels and one SF were used. For those cases, we only tested SF 7 and SF 12, as these two SFs have the highest and lowest data rates, respectively. In each graph, we give the percentage of lost packets duet to collisions, the percentage of packets received with the wrong payload CRC and the sum of both. In use cases involving only localization, which is one of the LPWAN recognized use cases [24], localization is done by identifying the gateways that received the packet and applying a triangulation method based on signal strength measurements. For such use cases, it does not matter if the payload CRC is incorrect. As soon as the header is received correctly and the SNR and RSSI values during header reception are available, localization can be achieved. For other use cases where correct reception of the payload of the data is important, the sum of both percentages will be taken into account to assess the network scalability. For this reason, we separated the curves of lost packets due to collisions and the wrong payload CRC received packets. For each case, we also give the average throughput in terms of frames per hour per device. As for IoT applications, it is important to know how many packets per hour can be sent on average, we used this KPI instead of throughput in terms of bps.

### 6.1. LoRa with 1% Radio Duty Cycle

For this set of simulations, we respected the radio duty cycle of 1% while targeting a maximal application layer update rate. As such, packets were transmitted as soon as possible, just after the waiting time imposed by the radio duty cycle mechanism plus a random factor.

**Figure 6:**
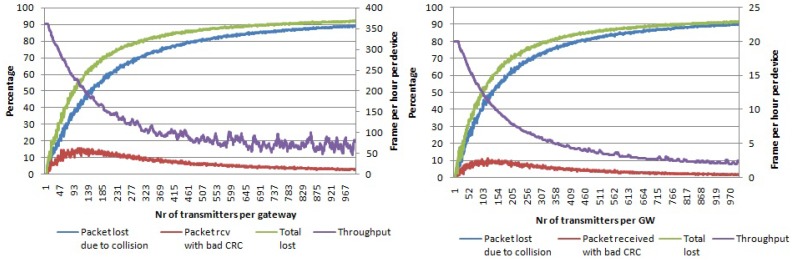
Percentage of packets lost due to collisions and percentage of packets received with the wrong payload CRC per number of transmitters per gateway. Average throughput per device. Single channel, single SF and payload size 20 bytes. (**left**) Using SF 7; (**right**) using SF 12.

In the first simulation set, we used a single channel and single SF. In Figure 6, the percentage of packets lost due to collisions and packets received with the wrong payload CRC is given for a payload size of 20 bytes and SF 7 and SF 12, respectively. When the number of transmitters per gateway is increased up to 1000, around 90% of all packets are collided. On the other hand, the packets received with the wrong payload CRC reach a peak of 15% (for SF 7) for 120 transmitters per gateway. After that, the graph decreases when increasing the number of nodes per gateway. This is the result of an increase in the number of collisions, meaning that there are fewer packets to be received with the wrong payload CRC. Regarding the average throughput per device, it decrease quickly with the number of nodes per gateway. Even though the percentage of lost packets is similar, it can be seen that with SF 7, we can send more packets per hour compared to SF 12 due to the higher data rates being used.

**Figure 7:**
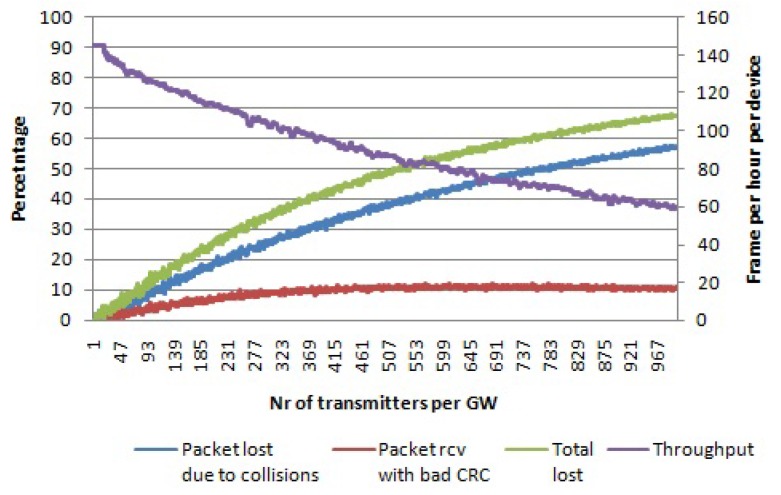
Percentage of packets lost due to collisions and percentage of packets received with the wrong payload CRC per number of transmitters per gateway. Average throughput per device. Payload size 20 bytes, single channel, multiple SFs.

By using multiple SFs (seven to 12), but only one physical channel, we increase the number of logical channels in use to six. In this case, the amount of collisions will decrease compared to the previous case. This is shown in Figure 7 where the total percentage of lost packets is now around 68% for 1000 nodes per gateway, which is lower compared to the single channel single SF case (SF 12) of 92%. In this case, where we have devices that use different SFs, the average throughput in terms of frames per hour per device will be between the highest one (350 frames per hour for SF 7 using 20 bytes payload frames) and the lowest one (20 frames per hour for SF 12 using 20 bytes payload frames). Furthermore, the throughput starts with a value that is smaller than in case when only SF 7 was used, but for a high number of devices in the network the throughput is similar to the case when only SF 7 was used and higher than when only SF 12 was used.

**Figure 8:**
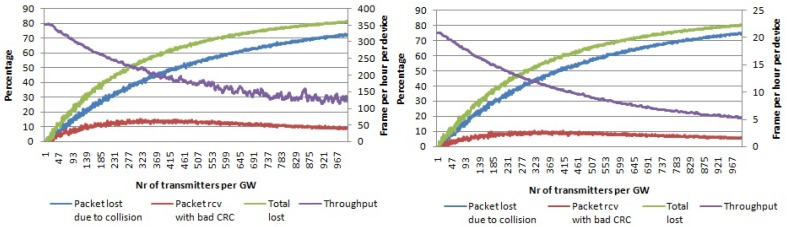
Percentage of packets lost due to collisions and the percentage of packets received with the wrong payload CRC per number of transmitters per gateway. Average throughput per device. Multiple channels, single SF and payload size 20 bytes. (**left**) Using SF 7; (**right**) using SF 12.

In the case of multiple channels and a single SF, the number of logical channels is the same as the number of physical channels, namely three. We performed simulations with SF 7 and SF 12. The graphs are shown in Figure 8, where it can be seen that for 1000 nodes per gateway around 75% (SF 12) of packets are lost due to collisions, which is lower than the case for single channel single SF, but higher than the case for single channel multiple SFs. Even the average throughput for 1000 devices per gateway is higher than in case when a single channel was used.

**Figure 9:**
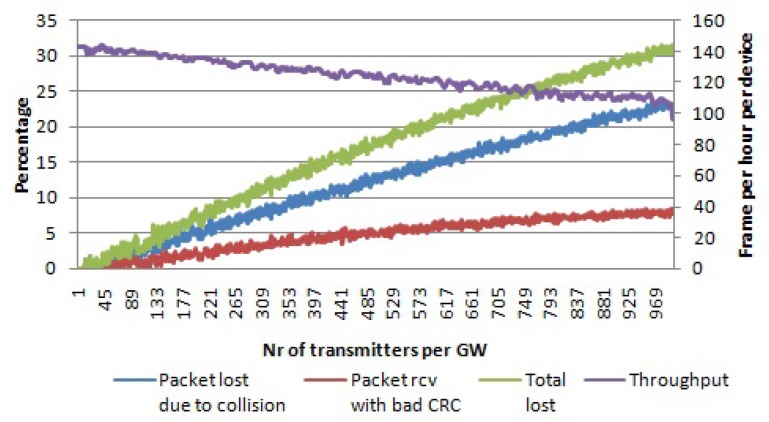
Percentage of packets lost due to collisions and the percentage of packets received with the wrong payload CRC per number of transmitters per gateway. Average throughput per device. Payload size 20 bytes, multiple channels, multiple SFs.

When an LoRaWAN network is able to optimally assign SFs to devices and make use of all mandatory channels, the network scalability and the number of nodes that it can serve can be maximized. When all of the SFs and all three data channels are used, then there are 18 non-interfering logical channels in total. Figure 9 shows the simulation results for this case. For 1000 nodes per gateway, 24% of the packets are lost due to collisions, while 8% of the packets are received with the wrong payload CRC. In total, 32% of packets are lost, which is much lower than in all other cases. Even throughput for a high number of devices per gateway is higher than in previous cases, as well.

**Figure 10:**
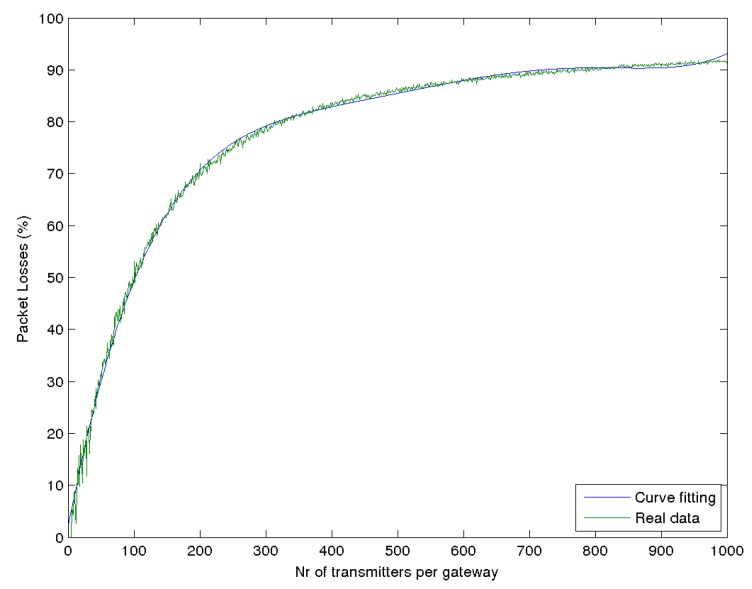
Curve fitting for single channel and SF 12.

Comparing Figure 6, Figure 7, Figure 8 and Figure 9, it can be seen that the packet losses scale with the degrees of freedom (the number of non-interfering logical channels). If you scale the X axis of Figure 9 by 18 or scale the X axis of the Figure 7 by six, we will get similar values as the ones we can get from Figure 6. Based on this observation, we can make use of a curve fitting method in order to express the curve of total packet loses for SF 12 from Figure 6 in a polynomial form. The fitted curve is shown in Figure 10. The function is:

(8) $$\begin{array}{c} f_{SCH\_SSF}\left(x\right)=1.1318\times 10^{-12}\times x^{5}-3.4342\times 10^{-9}\times x^{4}+4.0194\times 10^{-6}\times x^{3}\\ -0.0023\times x^{2}+0.6678\times x+1.7833;\;R^{2}=0.997\;x<1000 \end{array}$$

where *x* is the number of transmitters per gateway. All curves for the total percentage of lost packets from Figure 7, Figure 8 and Figure 9 can be constructed with the respective functions:

(9) $$f_{SCH\_MSF}\left(x\right)=f_{SCH\_SSF}\left(x/6\right);\;x<1000$$

(10) $$f_{MCH\_SSF}\left(x\right)=f_{SCH\_SSF}\left(x/3\right);\;x<1000$$

(11) $$f_{MCH\_MSF}\left(x\right)=f_{SCH\_SSF}\left(x/18\right);\;x<1000$$

where SCH is single channel, MCH is multiple channels, SSF single SF and MSF multiple SF.

### 6.2. Pure Aloha with 1% Radio Duty Cycle

The same simulations were run to check the pure Aloha scalability. Again, we respected the 1% duty cycle of the physical layer. The application layer update rate was not lower than the physical layer duty cycle, meaning that the packets were sent directly once the waiting time ended.

**Figure 11:**
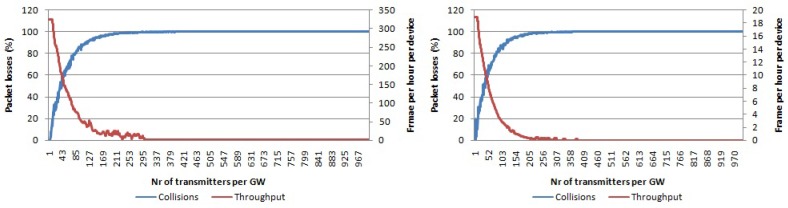
Percentage of packets lost due to collisions per number of transmitters per gateway using pure Aloha access. Average throughput per device. Single channel, single SF and payload size 20 bytes. (**left**) Using SF 7; (**right**) using SF 12.

In Figure 11, the packet loss percentage for the single-channel single-SF case using pure Aloha as the access technique is shown. We used SF 7 and SF 12. All traffic collides with only 200 nodes per gateway, which was not the case with LoRa due to its robust physical layer. Furthermore, the average throughput per device will go to zero for only 200 devices per gateway.

**Figure 12:**
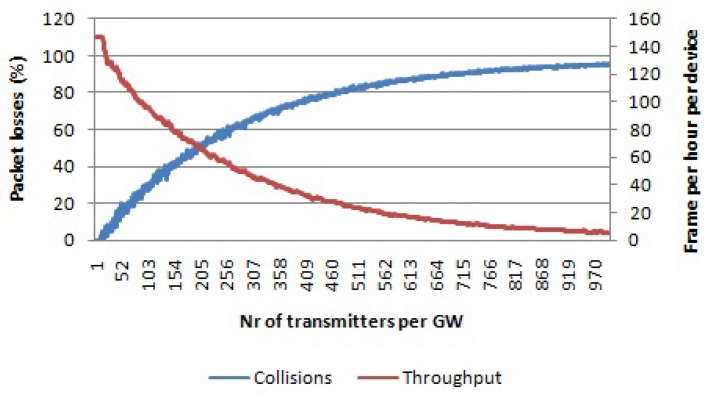
Percentage of packets lost due to collisions per number of transmitters per gateway using pure Aloha access. Average throughput per device. Payload size 20 bytes, single channel, multiple SFs.

By using multiple SFs, we can increase the number of non-interfering logical channels to six. The resulting number of collisions for an increasing number of nodes per gateway is shown in Figure 12. Compared with the case when using a single channel and single SF, scalability is now higher as for 200 nodes per transmitter, only around 50% of packets collided compared with 100% in the previous case. Average throughput per device is higher than zero even for more than 300 nodes per gateway in contrast with the previous case.

**Figure 13:**
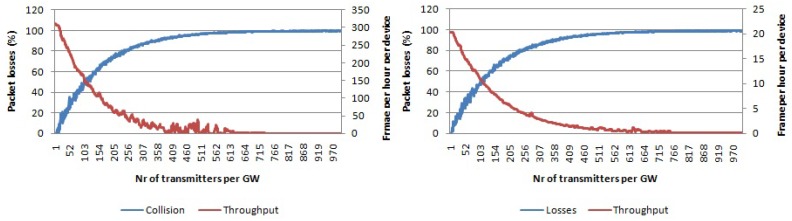
Percentage of packets lost due to collisions per number of transmitters per gateway using pure Aloha access. Average throughput per device. Multiple channels, single SF and payload size 20 bytes. (**left**) Using SF 7; (**right**) using SF 12.

In the case of multiple channels and single SF, we can only have three non-interfering logical channels. The collision graph for this case is given in Figure 13 for SF 7 and SF 12, respectively. Compared with the case using single channel and single SF, the scalability is higher as the graphs in Figure 13 increase slower than the graphs in Figure 11. Throughput goes to zero frames per hour per device for more than 620 nodes per gateway, which is lower than in case of single channel, multiple SF.

**Figure 14:**
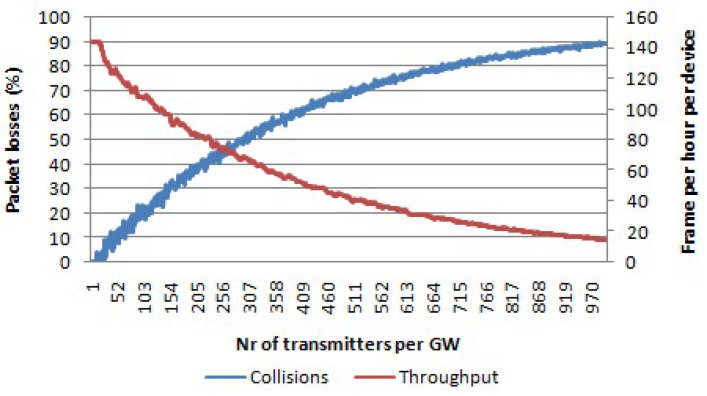
Percentage of packets lost due to collisions per number of transmitters per gateway using pure Aloha access. Average throughput per device. Payload size 20 bytes, multiple channels, multiple SFs.

When all non-interfering logical channels in the 868-MHz band are used, then the scalability will further increase. The collision graph using pure Aloha access for multiple channels and multiple SFs is given in Figure 14. For 1000 nodes per gateway, around 90% of packets collide, while the average throughput per device is around 20 frames per hour.

### 6.3. Comparison between LoRa and the Pure Aloha-Like Approach Simulations

**Figure 15:**
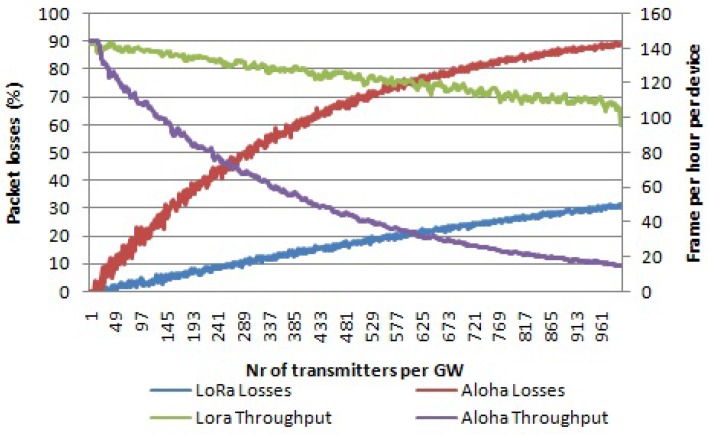
Comparison between LoRa and pure Aloha in terms of percentage of packets lost and average throughput per device. Multiple channels, multiple SFs and payload size of 20 bytes.

Comparing the pure Aloha approach with LoRa shows that LoRa performs better than pure Aloha in terms of scalability of the network. As discussed in the Related Work section, in [12], the authors claim that the LoRa performance is like pure Aloha performance in terms of collisions. However, they did not take into account the ability of the LoRa technology to receive packets under self-interference after the preamble and header is finished, as well as the capture effect during their simulations. In Figure 15, the collision graphs for both LoRa and the pure Aloha approach are given for the case of multiple channels and multiple SFs. We use six different SFs and three channels, so we have 18 logical channel is total. The channel bandwidth used is 125 kHz. It can be seen that LoRa clearly outperforms pure Aloha in terms of scalability. For 1000 devices per gateway, LoRa has fewer packet losses than pure Aloha, ∼32% and ∼90%, respectively. In this case, the graph for LoRa includes the percentage of packets lost due to collisions, as well as percentage of packets received with the wrong payload CRC, the curve for total packet losses percentage from Figure 9. The packet losses percentage for Aloha in Figure 15 is referred to Figure 13. The average throughput per device is ∼6 times more for LoRa than for pure Aloha, for a high number of devices per gateway (∼1000).

**Figure 16:**
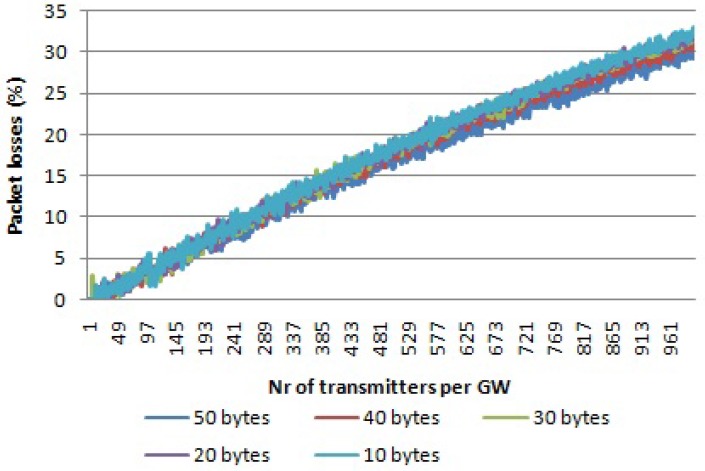
Comparison between different payload size. Multiple channels, multiple SFs.

We also checked the impact of the payload size on the scalability of the network. In Figure 16, the differences in packet loss percentage for different payload sizes for multiple channels and multiple SFs for LoRa is given. It can be seen that for a payload size of 50 bytes, the average number of collisions is lower for the same number of nodes per gateway. A longer time on air due to an increased packet size will be adjusted proportionally with the back-off times due the duty cycle, meaning that the collision rate is not affected. However, the recapture effect of LoRa is affected from the conclusion *i* of Section 4.3. For longer packets, the recapture window will be larger, making it possible to receive both interfered packets, increasing thus the reception ratio.

### 6.4. Simulations with a Lower Duty Cycle than the Physical Layer Duty Cycle

We are aware of the fact that many IoT applications have application layer duty cycles that are lower than 1%. Depending for which application the LoRaWAN network is used, it can have a different scalability in terms of the number of end devices that can be served. In [38], message transaction rates for different machine to machine communication applications are given. In addition to that, they show the node density for two cities (New York and Washington DC) for each application. Previously in [13], the authors calculated the number of end devices per LoRaWAN cell under the assumption of perfect synchronization of nodes and the usage of pure Aloha access. However, perfect synchronization between nodes is not possible, but as we saw in Section 4, LoRa gives better scalability than pure Aloha due to the robust physical layer.

Based on the data rates and end devices’ density from [38], we did simulations to determine the LoRa scalability for different IoT applications. The results are presented in Table 8. First of all, in some applications, the highest spreading factor cannot be used due to violation of the radio duty cycle by the message transaction period. We notice that for the traffic lights and roadway signs applications, we had to go down to SF 11 and SF 10, respectively. In cases where the highest spreading factor cannot be used, the total number of end devices is calculated based on the reduced coverage area imposed by the highest possible SF that can be used. From Table 8, we see that the scalability for different applications is different. Using only one gateway is not sufficient for most of the applications in a dense city like New York, taking into account the large number of devices for each sort of application, e.g., for the traffic lights application use case, 100% of the end devices can be served in a cell. However, for other considered applications, the percentage is quite low.

In any European city with end device density like in New York City, the scalability will increase if end devices will be configured to even make use of the other five optional channels at the 867-MHz sub-band. In such a case, we expect that the scalability will increase by 62% as we will have 48 non-interfering logical channels in total compared to only 18 mandatory ones that we used during our simulations. Furthermore, increasing the number of gateways will help in lowering the number of devices per cell, decreasing thus the self-interference inside one cell, as well.

## 7. Future Work

Next to uplink traffic, LoRAWAN can also handle downlink traffic. The same radio duty cycle rules also apply for gateways in the downlink communication. We believe that the impact of downlink traffic, as well as confirmed uplink messages will greatly affect the network scalability. Future work on the impact of MAC layer message packets in network scalability can be studied.

As the frequency bands that are used by LoRa can be used even by other technologies, future studies on the impact of other types of transmissions on LoRa communication can be done. In this case, different approaches can be taken, like using real nodes from other technologies to create interference and to check its impact on LoRa transmissions or to use SDR to emulate the interference from other technologies. Once the interference from other technologies is determined, then the impact on the network scalability can be calculated following the methodology presented in this paper.

Simulation with the system level simulator gives the opportunity to take into account the propagation models, multiple number of gateways, different traffic patterns, downlink traffic, etc. Implementation of modules in system level simulator, like ns-3, that allows this will further be studied.

## 8. Conclusions

In this paper, we assessed the single cell LoRaWAN network scalability in terms of the number of end nodes that can be served using a simulation model based on real measurements. First, we determined the impact of two concurrent LoRa transmissions on each other by using physical LoRaWAN end devices and a gateway in an RF shielded lab setup. A channel bandwidth of 125 kHz for devices was used. Even under concurrent transmission, one of the packets was received as soon as the last six symbols of preamble and header of the packet did not collide. Based on the outputs from this experiment, we created a simulation model to determine the network scalability for single-cell LoRaWAN.

We showed that LoRaWAN has better scalability than pure Aloha due to its robust physical layer. In the case of LoRaWAN, one of the packets could still go through even under collision conditions, which is not the case with the pure Aloha approach. We showed that one can send six-times more traffic with LoRaWAN than with pure Aloha in a single-cell LoRaWAN network for the same number of end devices per gateway when the 125-kHz channel bandwidth is used.

As different IoT applications have lower application duty cycles than the 1% radio duty cycle, we determined the number of devices that can be served by a single gateway in different IoT application use cases. We showed that in the case of traffic lights in a dense city, all of them can be served by LoRaWAN without requiring more gateways than what it is needed for coverage purposes. For other IoT application use cases, the scalability figures are quite low, and only low percentages of end devices can be served with acceptable total network losses.

## Figures and Tables

**Table 1:** Physical layer parameters for the measurement setup.

Parameter	Setting 1	Setting 2
Spreading Factor	12	7
Bandwidth	125 kHz	125 kHz
Code Rate	4/8	4/8
Explicit header	On	On
Channel	868.3 MHz	868.3 MHz
Payload CRC	On	On
Programmable preamble symbol length	8	14
Low Data Rate optimization	On	Off
Payload size	17 bytes	17 bytes
Equivalent bit rate	183.11 bps	3417.97 bps
On-air time	1712.13 ms	76.03
Preamble time duration	401.41 ms	18.69 ms
Symbol time	32.77 ms	1.02 ms

**Table 2:** Statistics for collided packets for the case when the interferer has the same RSSI as the interfered transmission for the first setting from Table 1. NA stands for Not Available.

**Shift in ms**	**100**		**200**		**300**		**400**		**500**		**600**	
**Transmitters**	Tx1	Tx2	Tx1	Tx2	Tx1	Tx2	Tx1	Tx2	Tx1	Tx2	Tx1	Tx2
**Packet lost due to** **collisions (%)**	2.2	100	4	100	3.2	100	3	100	4.4	100	2	100
**Packet received with** **BAD_CRC (%)**	25	NA	9	NA	4.4	NA	1	NA	7	NA	4.3	NA
**Average of SNR (dB)**	2	NA	2	NA	2.1	NA	2.2	NA	1.7	NA	1.8	NA
**Shift in ms**	**700**		**800**		**900**		**1000**		**1100**		**1200**	
**Transmitters**	Tx1	Tx2	Tx1	Tx2	Tx1	Tx2	Tx1	Tx2	Tx1	Tx2	Tx1	Tx2
**Packet lost due to** **collisions (%)**	3.4	100	2.4	100	0.8	100	0.8	100	2	100	2	100
**Packet received with** **BAD_CRC (%)**	3.5	NA	8.4	NA	4.3	NA	2.5	NA	5.4	NA	3.2	NA
**Average of SNR (dB)**	2.1	NA	2.4	NA	2.4	NA	2.5	NA	3	NA	3.4	NA
**Shift in ms**	**1300**		**1400**		**1500**		**1600**					
**Transmitters**	Tx1	Tx2	Tx1	Tx2	Tx1	Tx2	Tx1	Tx2				
**Packet lost due to** **collisions (%)**	1	100	2.2	84	1	24	2	5.2				
**Packet received with** **BAD_CRC (%)**	2.1	NA	0	0	2.1	1	4.5	0				
**Average of SNR (dB)**	4	NA	5	8.4	6.5	8.5	8.6	8.9				

**Table 3:** Statistics for collided packets for the case when the interferer has the same RSSI as the interfered transmission for the second setting from Table 1. NA stands for Not Available.

Shift in ms	5		10		20		30	
**Transmitters**	Tx1	Tx2	Tx1	Tx2	Tx1	Tx2	Tx1	Tx2
**Packet lost due to** **collisions (%)**	2.3	100	2.1	100	2.8	100	2.5	100
**Packet received with** **BAD_CRC (%)**	18	NA	7	NA	4	NA	5.2	NA
**Average of SNR (dB)**	3	NA	3.1	NA	3.2	NA	3.1	NA
**Shift in ms**	**40**		**50**		**60**		**70**	
**Transmitters**	Tx1	Tx2	Tx1	Tx2	Tx1	Tx2	Tx1	Tx2
**Packet lost due to** **collisions (%)**	2.1	100	2	100	0	100	0	0
**Packet received with** **BAD_CRC (%)**	3.1	NA	2.1	NA	2	NA	0	0
**Average of SNR (dB)**	4	NA	5	NA	8	NA	9.1	9.1

**Table 4:** Statistics for collided packets for the case when the interferer has a 12 dB higher RSSI than the interfered transmission for the first setting from Table 1. NA stands for Not Available.

**Shift in ms**	**100**		**200**		**300**		**400**		**500**		**600**	
**Transmitters**	Tx1	Tx2	Tx1	Tx2	Tx1	Tx2	Tx1	Tx2	Tx1	Tx2	Tx1	Tx2
**Packet lost due to** **collisions (%)**	100	100	100	100	100	100	100	0	62	50	2	100
**Packet received with** **BAD_CRC (%)**	NA	NA	NA	NA	NA	NA	NA	0	38	0	98	NA
**Average of SNR (dB)**	NA	NA	NA	NA	NA	NA	NA	10.3	1.01	9.7	1.3	NA
**Shift in ms**	**700**		**800**		**900**		**1000**		**1100**		**1200**	
**Transmitters**	Tx1	Tx2	Tx1	Tx2	Tx1	Tx2	Tx1	Tx2	Tx1	Tx2	Tx1	Tx2
**Packet lost due to** **collisions (%)**	0	100	0	100	0	100	0	100	0	100	0	100
**Packet received with** **BAD_CRC (%)**	100	NA	100	NA	100	NA	100	NA	100	NA	100	NA
**Average of SNR (dB)**	1.3	NA	1.1	NA	1.1	NA	1.7	NA	1.6	NA	1.7	NA
**Shift in ms**	**1300**		**1400**		**1500**		**1600**					
**Transmitters**	Tx1	Tx2	Tx1	Tx2	Tx1	Tx2	Tx1	Tx2				
**Packet lost due to** **collisions (%)**	0	100	0	48	0	0	0	0				
**Packet received with** **BAD_CRC (%)**	100	NA	99	2	73	0	1	0				
**Average of SNR (dB)**	5.1	NA	6	10.4	6.8	10.4	8.2	11.3				

**Table 5:** Statistics for collided packets for the case when the interferer has a 12 dB higher RSSI than the interfered transmission for the second setting from Table 1. NA stands for Not Available.

Shift in ms	5		10		20		30	
**Transmitters**	Tx1	Tx2	Tx1	Tx2	Tx1	Tx2	Tx1	Tx2
**Packet lost due to** **collisions (%)**	100	100	100	100	72	41	0	100
**Packet received with** **BAD_CRC (%)**	NA	NA	NA	NA	28	0	100	NA
**Average of SNR (dB)**	NA	NA	NA	NA	1.8	9.1	1.91	NA
**Shift in ms**	**40**		**50**		**60**		**70**	
**Transmitters**	Tx1	Tx2	Tx1	Tx2	Tx1	Tx2	Tx1	Tx2
**Packet lost due to** **collisions (%)**	0	100	0	100	0	100	0	0
**Packet received with** **BAD_CRC (%)**	100	NA	100	NA	100	NA	0	0
**Average of SNR (dB)**	2.1	NA	2.08	NA	6.19	NA	8.99	9.1

**Table 6:** Status of the packet from the interfered transmission for different cases from Figure 5.

Cases from Figure 5	Interferer RSSI >	Interferer RSSI ~	In Our Model
1st case	Lost	Lost	Lost
2nd case	Lost	Lost	Lost
3rd case	Lost	Lost	Lost
4th case	Lost	Received mostly correctly ∼90%	Lost
5th case	Lost	Received mostly correctly ∼90%	Lost
6th case	Lost	Received mostly correctly ∼90%	Lost
7th case	Received with the wrong CRC	Received correctly	Received with the wrong CRC or correctly based on RSSI

**Table 7:** Area covered by each spreading factor using the Okumura propagation model.

SF	Sensitivity Range (dBm) [28]	Distance Range (km)	Total Area (km${}^{2}$)	Percentage in Total Area
12	(−137, −135)	(8.13, 7.15)	47.02	22.65
11	(−135, −133)	(7.15, 6.28)	36.69	17.67
10	(−133, −130)	(6.28, 5.18)	39.58	19.07
9	(−130, −129)	(5.18. 4.86)	10.09	4.86
8	(−129, −124)	(4.86, 3.52)	35.25	16.99
7	(−124, −	(3.52, 0)	38.91	18.75
Total area covered by the gateway			207.54	

**Table 8:** LoRaWAN scalability for different IoT applications.

Applications	Transaction Message Period (s)	Payload Size (bytes)	Highest SF	No. of Nodes in 7 km Radius Cell in New York	No. of Nodes for Total Network Losses <10%	% of Nodes Served in One 7 km Radius Cell in New York
Home security	600	20	12	591,773	∼1400	0.24
Home appliances	86,400	8	12	1,775,319 ${}^{1}$	∼150,000	8.45
Roadway signs	30.03	1	10	9340 ${}^{2}$	∼650	6.95 ${}^{2}$
Traffic lights	59.88	1	11	152 ${}^{3}$	∼1200	100 ${}^{3}$
Credit machine in grocery	120.48	24	12	32,049	∼280	0.87

${}^{1}$ We took an average of 3 devices per house; ${}^{2}$ reduced area due to lower maximal SF, radius of 5.2 km; ${}^{3}$ reduced area due to lower maximal SF, radius of 5.5 km.

## Abbreviations

The following abbreviations are used in this manuscript:

CRC	Cyclic Redundancy Code
IoT	Internet of Things
LPWAN	Low Power Wide Area Networks
MAC	Medium Access Control
RSSI	Received Signal Strength Indicator
SF	Spreading Factor
SNR	Signal to Noise Ratio
